# Disparity in temporal and spatial relationships between resting-state electrophysiological and fMRI signals

**DOI:** 10.21203/rs.3.rs-3251741/v2

**Published:** 2024-01-10

**Authors:** Wenyu Tu, Samuel R. Cramer, Nanyin Zhang

**Affiliations:** 1 The Neuroscience Graduate Program, The Huck Institutes of the Life Sciences, Pennsylvania State University, University Park, USA; 2 Department of Biomedical Engineering, Pennsylvania State University, University Park, USA; 3 Center for Neural Engineering, Pennsylvania State University, University Park, USA; 4 Center for Neurotechnology in Mental Health Research, Pennsylvania State University, University Park, USA

**Keywords:** resting-state fMRI, electrophysiology, rat

## Abstract

Resting-state brain networks (RSNs) have been widely applied in health and disease, but their interpretation in terms of the underlying neural activity is unclear. To systematically investigate this cornerstone issue, here we simultaneously recorded whole-brain resting-state functional magnetic resonance imaging (rsfMRI) and electrophysiology signals in two separate brain regions in rats. Our data show that for both recording sites, band-specific local field potential (LFP) power-derived spatial maps can explain up to 90% of the spatial variance of RSNs obtained by the rsfMRI signal. Paradoxically, the time series of LFP band power can only explain up to 35% of the temporal variance of the local rsfMRI time course from the same site. In addition, regressing out time series of LFP power from rsfMRI signals has limited impact on the spatial patterns of rsfMRI-based RSNs. This disparity in the spatial and temporal relationships between resting-state electrophysiology and rsfMRI signals suggest that the electrophysiological activity alone does not account for all effects in the rsfMRI signal. To further interpret this disparity, we propose a model hypothesizing that a significant component in the rsfMRI signal is driven by electrophysiology-invisible neural activities that are active in neurovascular coupling. Temporally, this electrophysiology-invisible signal is weakly correlated to electrophysiology data. However, as signaling of these two types of neural activities are both constrained by the same anatomical backbone, they can generate similar RSN spatial patterns. These data and the model provide a new perspective of our interpretation of RSNs.

## Introduction

Sophisticated brain function requires coordinated activities from separate brain regions, which collectively constitute functional brain networks. Functional brain networks in humans and animals are predominantly studied using the method of resting-state functional magnetic resonance imaging (rsfMRI), which quantifies the synchronization of brain-wide spontaneous blood-oxygen-level dependent (BOLD) signals, and such networks are termed as resting-state brain networks (RSNs).

Although BOLD-derived RSNs have been widely applied in health and disease, their relationship to the underlying neural activity is not fully understood. This is a fundamental issue, because the BOLD signal is known to not directly, but indirectly reflect neural activity via the accompanied hemodynamic/metabolic changes, a mechanism called neurovascular coupling (NVC). Although tight NVC has been repeatedly demonstrated when neural activities are evoked by explicit external stimulation (Goense and Logothetis, 2008; Logothetis et al., 2001; Nemoto et al., 2004), this relationship in the resting state remains elusive. On the one hand, substantial evidence demonstrates that the spatial patterns of most RSNs well replicate known functional systems and activation patterns elicited by various tasks (Biswal et al., 1995; Glasser et al., 2016; Hampson et al., 2002; Lowe et al., 1998; Smith et al., 2009), as well as patterns of structural networks (Andrews-Hanna et al., 2010; Greicius et al., 2009; Lowe et al., 2008). In addition, alterations in RSNs are reported in various brain disorders that correspond to neuropathophysiological changes (Buckner et al., 2009), collectively suggesting that RSNs must have strong neural basis. On the other hand, time series of the resting-state electrophysiological signal often has fairly low prediction power of the corresponding rsfMRI time series (Mateo et al., 2017; Scholvinck et al., 2010; Winder et al., 2017), and some studies even show a disconnect between neural activity and hemodynamic signals in certain conditions (Maier et al., 2008; Zhang et al., 2019). In addition, different studies reported mixed electrophysiology correlates of the rsfMRI signal across local field potential (LFP) bands in a wide frequency range, from infraslow signal (Hiltunen et al., 2014; Pan et al., 2013), low-frequency delta/sub-delta band signal (He et al., 2008; Lu et al., 2007) to high-frequency gamma band signal (Bastos et al., 2015; Foster et al., 2015; Keller et al., 2013; Mukamel et al., 2005; Nir et al., 2008) and spiking activity (Mukamel et al., 2005), which indicate that the rsfMRI signal may reflect different aspects of neural activity. Taken together, it is far from being clear how RSNs and rsfMRI are related to spontaneous neural activity (Laufs et al., 2003; Liu et al., 2011; Mantini et al., 2007), and lack of such knowledge represents a major knowledge gap in our ability to interpret functional brain networks.

To address this critical issue, we systematically examined the role of electrophysiology activity in determining specific spatiotemporal patterns of BOLD-based RSNs. Along with whole-brain rsfMRI, electrophysiology signals were simultaneously recorded in the primary motor cortex (M1) and anterior cingulate cortex (ACC) in rats under both light sedation and awake conditions. M1 and ACC were selected given their distinct role in sensorimotor function and integrative cognition, respectively. Our data show that in both light-sedation and awake states, the gamma band power-derived RSN spatial patterns highly resemble BOLD-derived RSNs for both recording sites, and lower frequency band-derived RSNs exhibit inversed spatial patterns, both indicating strong neural underpinning of RSNs. Paradoxically, at both recording sites, the time course of band-limited LFP powers display considerably lower temporal correlations with the corresponding time course of local BOLD signals. Further, regressing out the gamma band power or powers of all LFP bands has limited effects on the spatial pattern of BOLD-derived RSNs, collectively suggesting LFP powers only play a partial role in the local rsfMRI signal. This disparity in spatial and temporal relationships between resting-state BOLD and electrophysiology signals indicate that there might be an electrophysiology-invisible component of brain activities that have major contributions to the rsfMRI signal and RSNs.

## Results

**Figure 1.**
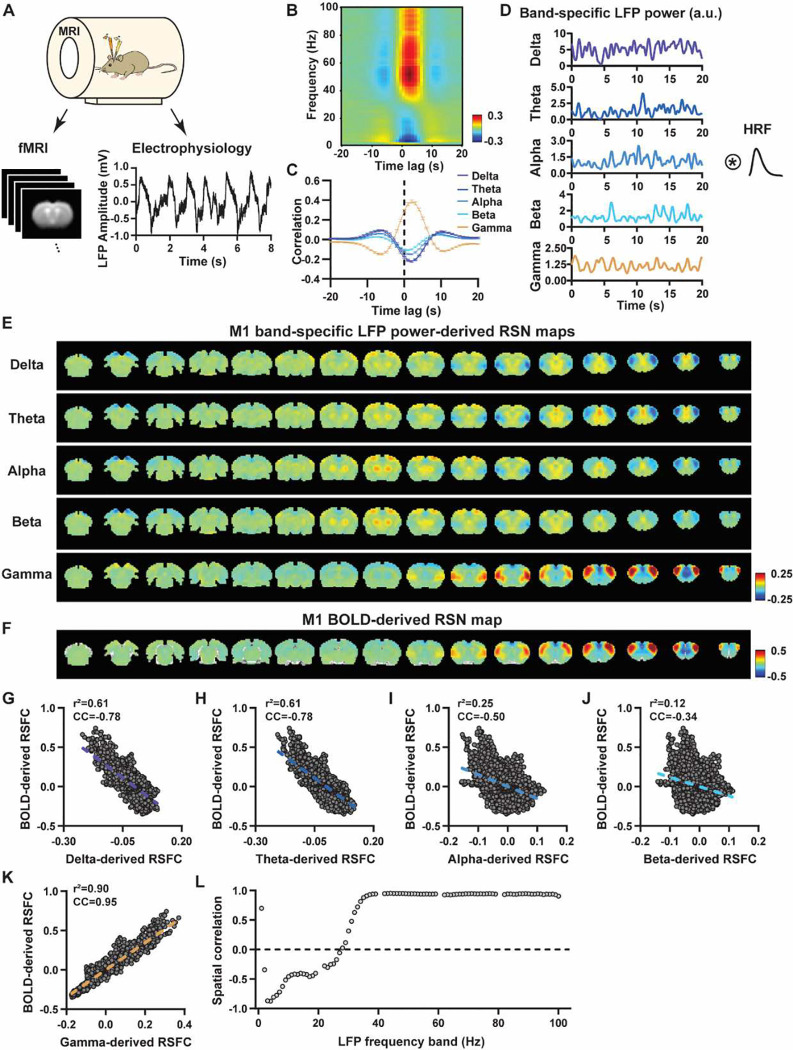
LFP and rsfMRI signals in the M1 derive highly consistent RSN spatial patterns in lightly sedated rats. **A**) Simultaneous acquisition of whole-brain rsfMRI and electrophysiological signals in the M1 and ACC. **B**) Cross correlations between the rsfMRI signal and LFP power in the M1 (0.1–100 Hz, band interval: 1Hz, lag range: −20 – 20 s. **C)** Cross correlations between the rsfMRI signal and powers of individual LFP bands in the M1. Bars: SEM. **D**) Exemplar powers of individual LFP bands. Convolving powers of individual LFP bands with a rodent-specific hemodynamic response function (HRF) generates the corresponding LFP-predicted BOLD signals. **E**) M1 band-specific LFP power-derived RSN maps, obtained by voxel-wise correlating the LFP-predicted BOLD signal for each band with BOLD signals of all brain voxels. **F**) M1 BOLD-derived RSN map (i.e. M1 seedmap), obtained by voxel-wise correlating the regionally averaged BOLD time course of the seed (i.e. M1) with BOLD time courses of all brain voxels. **G-K**) Spatial similarity between the M1 BOLD-derived RSN map and the M1 LFP-derived RSN map for each band, quantified by their voxel-to-voxel correlations (G: delta, CC = −0.78; H: theta, CC = −0.78; I: alpha, CC = −0.5; J: beta, CC = −0.34; K: gamma, CC = 0.95). **L**) Spatial correlations between the M1 BOLD-derived RSN map and RSN maps derived by individual 1-Hz bands in the full LFP spectrum.

To systematically analyze the spatiotemporal relationship between resting-state electrophysiology and fMRI signals, we simultaneously recorded the whole-brain rsfMRI and electrophysiology signals in the M1 and ACC in rats in both light-sedation and awake states (Fig. 1A). The placement of implanted electrodes in the M1 and ACC was verified using T2-weighted structural images (Fig. S1). Raw electrophysiology data were initially preprocessed to remove MR artifacts using a template regression approach (Tu and Zhang, 2022). The LFP was obtained by bandpass filtering preprocessed electrophysiology data in the frequency range of 0.1 – 300 Hz. An example of denoised LFP is shown in Fig. 1A and Fig. S2. The band-specific LFP power was generated using a conventional LFP band definition (delta: 1–4 Hz, theta: 4–7 Hz, alpha: 7–13 Hz, beta: 13–30 Hz, gamma: 40–100 Hz). Figs. 1B&C show cross correlations of the LFP power and BOLD signal in the M1 across the LFP spectrum (Fig. 1B, 1-Hz band interval) and for individual LFP bands (Fig. 1C), indicating that gamma band power is positively correlated with the BOLD signal, while lower-frequency bands display negative peak correlations with the BOLD signal. The lag of the BOLD signal is approximately 2 sec for all bands, consistent with the hemodynamic response function (HRF) delay previously reported in rodents (Scholvinck et al., 2010; Winder et al., 2017).

### LFP and rsfMRI signals derive consistent RSN spatial patterns in lightly sedated rats.

**Figure 2.**
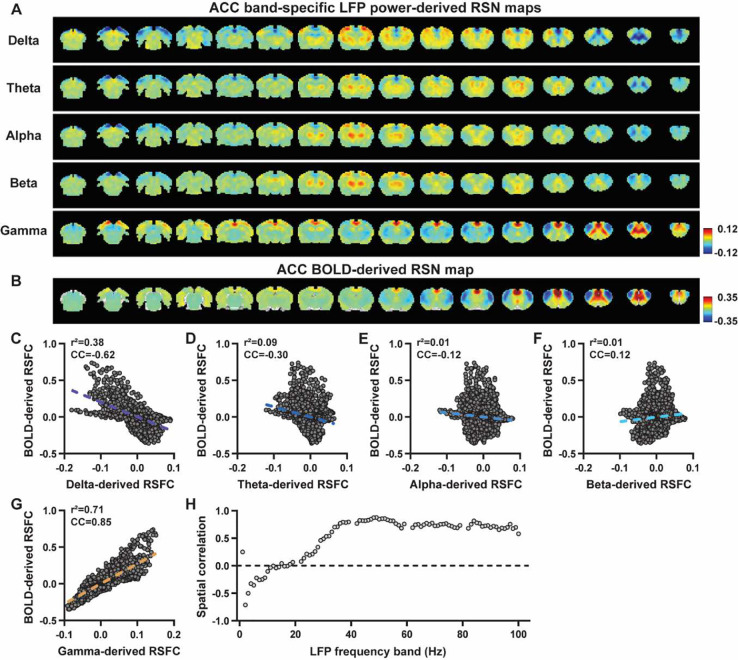
LFP and rsfMRI signals in the ACC derive highly consistent RSN spatial patterns in lightly sedated rats. **A**) ACC band-specific LFP power-derived RSN maps, obtained by voxel-wise correlating the LFP-predicted BOLD signal for each band with BOLD signals of all brain voxels. **B**) ACC BOLD-derived RSN map (i.e. ACC seedmap), obtained by voxel-wise correlating the regionally averaged BOLD time course of the seed (i.e. ACC) with BOLD time courses of all brain voxels. **C-G**) Spatial similarity between the ACC BOLD-derived RSN map and the ACC LFP-derived RSN map for each band, quantified by their voxel-to-voxel spatial correlations (C: delta, CC = −0.62; D: theta, CC = −0.30; E: alpha, CC = −0.12; F: beta, CC = 0.12; G: gamma, CC = 0.85). **H**) Spatial correlations between the ACC BOLD-derived RSN map and RSN maps derived by individual 1-Hz bands in the full LFP spectrum.

We first examined the spatial relationship between brain-wide rsfMRI signals and frequency band-specific LFP powers in lightly sedated rats. For each recording site, its BOLD-derived RSN is obtained as the seedmap, calculated by voxel-wise correlating the regionally averaged BOLD time series of the seed (M1 in Fig. 1F and ACC in Fig. 2B) with BOLD time series of individual brain voxels. This map is conventionally defined as the resting-state functional connectivity (RSFC) pattern for the seed region. To determine to what extent this RSN can also be derived using the LFP signal recorded from the same location (M1 or ACC), we convolved power of each LFP band with an rodent-specific HRF (Fig. 1D, (Tong et al., 2019)) to generate the LFP band-predicted BOLD signal, which was then voxel-wise correlated with the brain-wide rsfMRI signal, yielding the LFP band-derived RSN map (Fig. 1E, Fig. 2A). We found that the gamma band power-derived RSN map is spatially highly consistent with the corresponding BOLD-derived RSN map (i.e. seedmap). For M1, the voxel-to-voxel Pearson correlation coefficient (CC) between the mean gamma-derived RSN map (Fig. 1E) and mean BOLD-derived RSN map (Fig. 1F) is 0.95 (Fig. 1K, R^2^ = 0.90), indicating 90% of the variance in the M1 BOLD-derived RSN map can be explained by the gamma-derived map. The spatial maps generated by lower-frequency bands are inversely correlated with the M1 BOLD-derived RSN maps, with a trend of more negative spatial CC in lower-frequency bands (Figs. 1E–J, delta: CC = −0.78; theta: CC = −0.78; alpha: CC = −0.5; beta: CC = −0.34), consistent with the LFP-BOLD cross correlations for these bands shown in Fig. 1B–C. These relationships are repeatable in the ACC (Figs. 2A–G, delta: CC = −0.62; theta: CC = −0.3; alpha: CC = −0.12; beta: CC = 0.12; gamma: CC = 0.85). These data indicate that the spatial patterns of BOLD-based RSNs can be reliably obtained using the band-specific LFP signal.

To examine whether these findings actually result from the specific frequency cutoffs we adopted for any LFP band, we repeated the analysis for all individual 1-Hz bands in the full LFP spectrum. Our data again show a gradual transition from negative to positive spatial correlations in LFP-derived RSN maps with the corresponding BOLD-derived RSN map as the LFP signal goes from low to high frequencies (Fig. 1L for M1; Fig. 2H for ACC). Furthermore, as a control analysis, the gamma band power in the ACC was temporally shuffled, convolved with the HRF and the correlation map was recalculated (Fig. S3). In this case the spatial pattern observed in Fig. 2A disappeared, suggesting the LFP-derived spatial patterns are indeed specifically related to the LFP signal, instead of an artifact of HRF. We also confirmed that all our results are not sensitive to the rsfMRI data preprocessing step of global signal regression (Figs. S4-S5).

These data collectively indicate the BOLD-derived RSNs can be reliably replicated using LFP power from the same site in lightly sedated rats, suggesting the critical involvement of neural activity in RSN spatial patterns.

### Temporal correlation between LFP power and local rsfMRI signal is significant but considerably weaker.

Given the high reliability of the gamma power in determining spatial patterns of BOLD-based RSNs, it is reasonable to assume the HRF-convolved gamma power ought to reliably predict the rsfMRI time series from the same location. To test this hypothesis, for each recording site temporal correlations of local rsfMRI time series and HRF-convolved LFP powers were calculated for individual scans, and resulting correlation values were then averaged across scans. Surprisingly, the local rsfMRI signal displays considerably weaker temporal correlations with LFP powers. In the M1, the LFP-BOLD temporal correlations gradually change from negative to positive as the LFP signal goes from low to high frequencies—the same trend observed in spatial correlations (Figs. 1E–K), with, however, significant but considerably lower absolute magnitude in CC values (one sample t-tests, delta: CC = −0.20, p = 2.1 × 10^-57^; theta: CC = −0.19, p = 7.1 × 10^𢈒62^; alpha: CC = −0.11, p = 1.2 × 10^−42^; beta: CC = −0.06, p = 8.4 × 10^−15^; gamma: CC = 0.37; p = 2.1 × 10^−58^; number of scans = 159). The same results are observed in the ACC (one sample t-tests; delta: CC = −0.13, p = 1.9 × 10^−28^; theta: CC = −0.04, p = 8.4 × 10^−7^; alpha: CC = −0.03, p = 6.0 × 10^−5^; beta: CC = −0.01, p = 0.1; gamma: CC = 0.18, p = 6.7 × 10^−42^; number of scans = 172). We also confirmed that lower temporal correlations are not due to the HRF used (Fig. S6).

**Figure 3.**
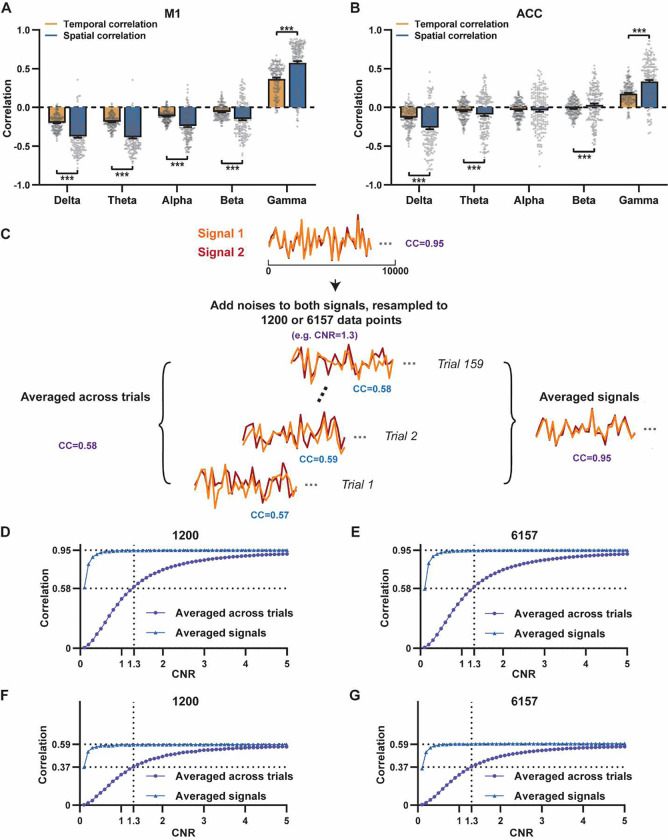
Disparity in spatial and temporal correlations persists after controlling for the noise effect. **A**, **B**) Comparison of scan-wise spatial and temporal correlations (paired t-tests across individual scans. ***:p<0.005); **C-G**) Simulation to evaluate factors that affect apparent correlation values including contrast-to-noise ratio (CNR) and the number of data points **C**) Two fixed signals with the true correlation coefficient of 0.95 are simulated (10000 data points) with random noise at a given CNR level being added. This process is repeated 159 times (i.e. # of scans in our study) for each CNR level. At each CNR, the CC was calculated either based on the averaged signals from all 159 trials (i.e. denoised data, triangle dots in D-G), or on signals of individual trials (i.e. with-noise data, round dots in D-G) before the resulting correlations are averaged across trials. **D**) Simulated signals resampled to 1200 data points (equal to the number of time points used to calculate temporal correlations). **E**) Simulated signals resampled to 6157 data points (equal to the number brain voxels used to calculate spatial correlations). Importantly, we can replicate the difference between true (R = 0.95) and apparent (R = 0.58) correlations obtained from denoised data and with-noise data, respectively, when CNR = 1.3. Therefore, we estimate that the CNR of our BOLD data is ~1.3. **F,G**) The same process as C-E with the true correlation of 0.59. This true correlation value is obtained by iteratively setting different true correlation values and searching for the one that provides the trial-wise apparent correlation of 0.37 (as measured by the gamma-BOLD temporal correlation in our real data, Figs. 3A) at CNR = 1.3. **F**) Simulated signals resampled to 1200 data points. **G**) Simulated signals resampled to 6157 data points.

Notably, one difference in our calculation of spatial and temporal correlations may contribute to the disparity in their CC values. In the calculation of spatial correlations, we first scan-wise generated LFP- and BOLD-derived RSN maps, and these maps were then averaged within each group before the spatial correlations were calculated using two averaged maps (Figs. 1–2). On the other hand, temporal correlations were first calculated for individual scans before the resulting correlation values were averaged across scans, as averaging time courses first would diminish the actual signal due to the semi-random nature of spontaneous brain activities. As a result, the variance in averaged RSN spatial maps might be lower than the variance in time series of individual scans, which can result in higher apparent spatial correlations than temporal correlations. To control for this factor, we also calculated spatial correlations between LFP- and BOLD-derived RSN maps for individual scans, and then averaged the corresponding correlation values across scans. Like scan-wise temporal correlations, scan-wise spatial correlations are significant for all LFP bands (one sample t-tests; in the M1, delta: CC = −0.37, p = 4.9 × 10^−52^; theta: CC = −0.39, p = 3.9 × 10^−62^; alpha: CC = −0.24, p = 5.8 × 10^−37^; beta: CC = −0.15, p = 3.9 × 10^−15^; gamma: CC = 0.58, p= 1.0 × 10^−56^; in the ACC, delta: CC = −0.26, p = 2.6 × 10^−27^; theta: CC = −0.09, p = 1.7 × 10^−6^; alpha: CC = −0.04, p = 0.04; beta: CC = 0.03, p = 0.06; gamma: CC = 0.33; p=5.4 × 10^−40^). Comparisons of scan-wise spatial and temporal correlations (Figs. 3A–3B) indicate that even after controlling for the variance level, the magnitude of spatial correlations are still appreciably higher than that of temporal correlations for all bands (paired t tests across individual scans; in the M1, delta: p = 1.01 × 10^−35^; theta: p = 3.74 × 10^−50^; alpha: p = 3.54 × 10^−25^; beta: p = 1.18 × 10^−12^; gamma: p= 7.02 × 10^−42^; in the ACC, delta: p = 6.73 × 10^−21^; theta: p = 5.75 × 10^−5^; alpha: p = 0.74; beta: p = 7.34× 10^−5^; gamma: p=3.43 × 10^−29^).

Given that the gamma power-derived RSN map can explain ~90% of spatial variance of the BOLD-derived RSN map (Fig. 1K, R = 0.95, R^2^ = 0.90) when noise is diminished by averaging RSN maps across scans, we ask how much variance of the local BOLD time series the gamma power can explain without significant influence of noise. Although we cannot directly average rsfMRI/electrophysiology time courses across scans to reduce the noise level like we did when calculating spatial correlations, we can still estimate the true LFP-BOLD temporal correlation by quantitatively assessing the effect of noise on correlation values. To this end, we leveraged the difference between the spatial correlation of averaged M1 RSN maps (i.e. termed as denoised data, R = 0.95, Fig. 1K) and that of unaveraged RSN maps (i.e. termed as with-noise data, R = 0.58 (Fig. 3A, gamma band power). Using this difference, we simulated two fixed signals with the true correlation of 0.95, and by adding different noise to the signals we determined at what noise level the apparent correlation of the two signals became 0.58 (Fig. 3C). Specifically, in each trial random noise at a defined contrast-noise-level (CNR) level was added to the simulated signals, and this process was repeated 159 times (i.e. equal to # of scans in our study) for a given CNR level. At each CNR, the correlation was calculated either based on the averaged signals from all 159 trials (i.e. simulating denoised data, Fig. 3C), or on signals of individual trials (i.e. simulating with-noise data) before the resulting correlations were averaged across trials.

As expected, lower CNR gives lower apparent trial-wise correlation values. Interestingly, we found that the trial-wise apparent correlation of 0.58 with the true correlation of 0.95 corresponds to the CNR at 1.3 (Fig. 3D–E), consistent with the CNR of BOLD contrast reported in the literature (Atkinson et al., 2008). At this CNR level, we estimated that the true BOLD-LFP temporal correlation in the M1 should be ~0.59 (R^2^ = 0.35, Fig. 3F–G) when the apparent correlation is 0.37 as measured by the gamma-BOLD temporal correlation in our real data (Fig. 3A). These data indicate the gamma power time information can only explain a minor portion (up to ~35%) of the temporal variance in the BOLD time series even after removing the noise effect. These results are consistent with previous reports of relatively weak temporal correlations between gamma power and hemodynamic signals at rest obtained using different imaging modalities (Scholvinck et al., 2010; Winder et al., 2017). Our simulation also indicates the difference in the number of data points (1200 in temporal correlation calculation vs 6157 in spatial correlation calculation) has a negligible influence on correlation values (Fig. 3E–G).

### Regressing out LFP powers has limited impact on RSN spatial patterns.

**Figure 4.**
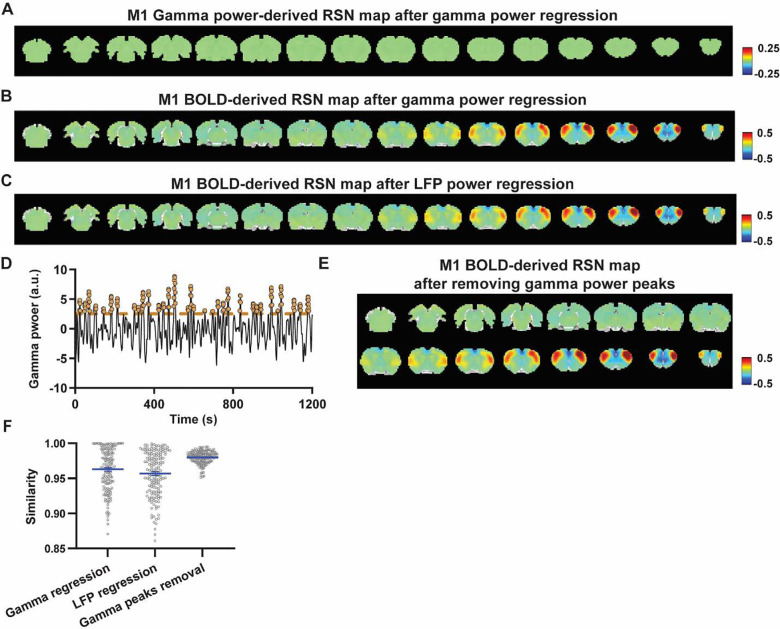
Impact of removing the electrophysiology signal on M1 BOLD-derived RSN spatial patterns. **A**) Gamma power-derived RSN map after the HRF-convolved gamma power in the M1 is regressed out from rsfMRI signals of all brain voxels. **B**) M1 BOLD-derived RSN map (i.e. M1 seedmap) after the HRF-convolved gamma power is voxel-wise regressed out from rsfMRI signals. **C**) M1 BOLD-derived RSN map after all five LFP band powers were voxel-wise regressed out from rsfMRI signals using soft regression. **D**) Peaks of HRF-convolved gamma power in one representative scan. **E**) M1 BOLD-derived RSN map after 15% rsfMRI time points corresponding to gamma peaks were removed. **F**) Spatial similarity of M1 BOLD-derived RSN maps before and after gamma power regression, regression of all LFP band powers or gamma peak removal.

**Figure 5.**
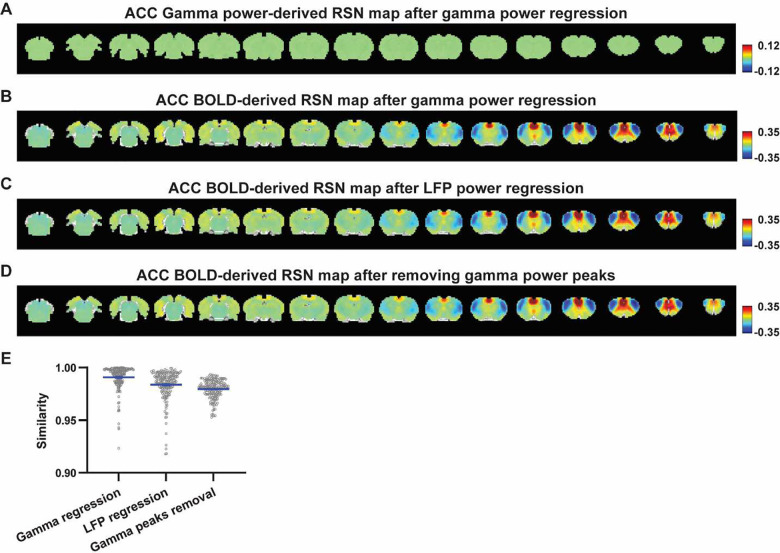
Impact of removing the electrophysiology signal on ACC BOLD-derived RSN spatial patterns. **A**) Gamma power-derived RSN map after the HRF-convolved gamma power in the ACC is regressed out from rsfMRI signals of all brain voxels. **B**) ACC BOLD-derived RSN map (i.e. ACC seedmap) after the HRF-convolved gamma power is voxel-wise regressed out from rsfMRI signals. **C**) ACC BOLD-derived RSN map after all five LFP band powers were voxel-wise regressed out from rsfMRI signals using soft regression. **D**) ACC BOLD-derived RSN map after 15% rsfMRI time points corresponding to gamma peaks were removed. **E**) Spatial similarity of ACC BOLD-derived RSN maps before and after gamma power regression, regression of all LFP band powers or gamma peak removal.

Given the lower predictive value of the LFP power on the local rsfMRI signal, we ask to what extent the temporal information of LFP powers affect the RSN spatial patterns. The M1 (or ACC) gamma-band power, after convolving with HRF, was linearly regressed out from th rsfMRI signals of all brain voxels. As expected, the spatial patterns of gamma power-derived RSN maps observed in Figs. 1E & 2A disappeared after the regression (Figs. 4A, 5A). However, this regression process minimally altered M1/ACC BOLD-derived RSN maps (Figs. 4B, 5B). This result remains the same when the powers of all LFP bands were voxel-wise regressed out from rsfMRI signals using soft regression (Figs. 4C, 5C). Soft regression was used as it prevents the multicollinearity issue in the regression model resulting from potential correlations between LFP bands, as this method only allows unique components in five LFP bands to be regressed out.

To examine whether the regression process is disproportionally dominated by time points with the largest LFP amplitude (i.e. outliers), we recalculated the M1/ACC BOLD-derived RSN maps after removing rsfMRI volumes corresponding to peaks in the M1/ACC gamma power (i.e. time points with the signal amplitude above 85 percentile in HRF-convolved gamma power of each scan, Figs. 4D, 4E & 5D). The spatial similarities between the BOLD-derived RSN maps before and after gamma power regression, all band power regression, or peak removal are summarized in Figs. 4F & 5E, showing that the removal of gamma power has limited impact on the M1/ACC RSN maps.

Considering that regression is a linear process, to control for the possible nonlinear relationship between band-specific LFP powers and the rsfMRI signal, we voxel-wise calculated the mutual information between band-limited LFP powers and rsfMRI signals for all brain voxels (Fig. S7). The data show limited mutual information between any band-specific power and voxel-wise rsfMRI signals, indicating that the nonlinear component between BOLD and electrophysiological signals does not play a major role in RSN spatial patterns. These data collectively indicate the temporal fluctuations of LFP have limited effects on BOLD-derived RSN spatial patterns.

### Disparity in temporal and spatial correlations is consistent in different physiologic states.

To determine whether the disparity between temporal and spatial correlations of resting-state LFP and fMRI signals we observed is a specific phenomenon under anesthesia or can be generalized to different physiologic states, we concurrently collected electrophysiology and rsfMRI data in awake rats. Despite the drastic change in the physiologic state, we again observed higher spatial correlations between LFP-derived maps (Fig. S8A) and the BOLD-derived RSN map (Fig. S8B) in the M1, which gradually changed from negative correlations in low-frequency bands to positive correlations in high-frequency bands, revealed both in conventionally defined bands (Figs. S8C-G) and 1-Hz bands (Fig. S8H). This trend is generally maintained in the ACC, although the spatial correlations in low-frequency bands are diminished (Fig. S9). Similar to the results in Fig. 3, weaker but significant temporal correlations between the rsfMRI signal and HRF-convolved gamma band power were observed in unanesthetized rats (one sample t-tests; for M1, delta: CC = −0.05, p = 2.3 × 10^−4^; theta: CC = −0.06, p = 4.7 × 10^−6^; alpha: CC = −0.06, p = 4.6 × 10^−5^; beta: CC = −0.032, p = 3.0 × 10^−3^; gamma: CC = 0.04, p= 0.02; for ACC, delta: CC = 0.02, p = 0.29; theta: CC = 0.0009, p = 0.96; alpha: CC = −0.02, p = 0.31; beta: CC = 0.006, p = 0.66; gamma: CC = 0.10, p= 9.52 × 10^−7^; number of scans = 50). Scan-wise comparison between temporal and spatial correlations are shown in Fig. S10. Overall, we found that the magnitudes of both spatial and temporal correlations are generally lower in the awake state compared to the light sedation state, likely due to larger variances from motion and/or other physiological fluctuations, as well as a much lower number of scans (50 awake scans, 159 light-sedation scans for M1 and 172 light-sedation scans for ACC), but the pattern of lower temporal but higher spatial correlations between gamma power and the rsfMRI signal remains consistent.

Regressing out gamma-band power or powers of all LFP bands again did not significantly alter the BOLD-derived RSN maps for both ACC and M1 in awake rats (Fig. S11). Taken together, these results show that all major findings in lightly anesthetized rats shown above can be replicated in unanesthetized rats, suggesting that these results are not specifically due to the effects of anesthesia.

### Ongoing rsfMRI signal could be contributed by electrophysiology-invisible brain activities.

**Figure 6.**
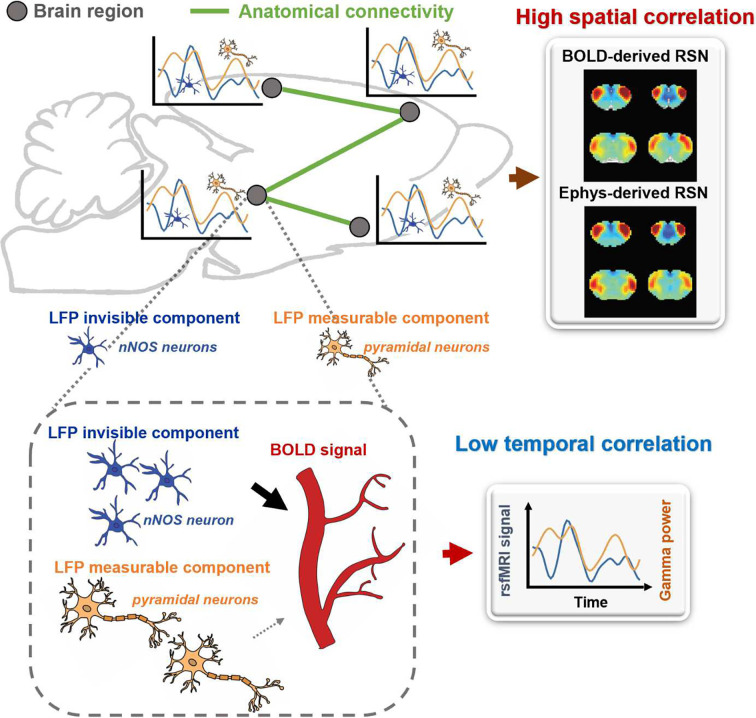
A proposed theoretic model that can explain the disparity in spatial and temporal correlations between resting-state electrophysiology and fMRI signals.

Our data reveal that the LFP signal can derive RSN patterns that can explain nearly all spatial variance in BOLD-based seedmaps. On the other hand, the temporal information of the LFP signal only explains a minor portion of the local BOLD time series and minimally impacts BOLD-based RSN spatial patterns. To reconcile this apparent contradiction, here we propose a theoretical model that can possibly explain this apparent paradox, described as follows:

Brain activities include components that can be measured by electrophysiology and components that are electrophysiology invisible. Electrophysiology-invisible brain activities, such as activities of nNOS neurons and astrocytes, are actively involved in NVC, and can play a major role in the rsfMRI signal. At the resting state, these two components are not necessarily synchronized, leading to low temporal correlations between electrophysiology and rsfMRI signals. Another factor that can contribute to low LFP-BOLD temporal correlations is neuromodulations from distal modulator nuclei (e.g. locus coerulues and/or basal forebrain), which have strong vasoactive effects but may not commensurately affect the electrophysiology activity. Meanwhile, signaling of electrophysiology activities as well as that of electrophysiology-invisible, and thus major BOLD activities are both constrained by the same anatomical pathways, allowing the two signals to separately generate similar RSN spatial patterns which can reflect both direct and indirect anatomical connectivity. The model is summarized in Figs. 6 and S13. More details of the model will be discussed in the next section.

## Discussion

Although BOLD-derived RSNs have been widely investigated in multiple species (Damoiseaux et al., 2006; Lu et al., 2012; Mantini et al., 2011) and applied in health and disease, the interpretation of interareal spontaneous BOLD synchronization is not fully comprehended. To gain more insight into this issue, here we systematically analyzed electrophysiology and rsfMRI signals simultaneously recorded in lightly anesthetized and unanesthetized animals. We found that both electrophysiology and rsfMRI signals can derive highly consistent brainwide RSN patterns, but the temporal information of the LFP signal only minorly contributes to the BOLD time series. These paradoxical results, as well as those from literature studies can possibly be reconciled by the theoretical model we propose. These data and the model provide a new perspective for our interpretation of the neural basis underlying the resting-state BOLD signal.

The spatial correspondence between BOLD- and electrophysiology-derived RSNs has been repeatedly reported using multiple methodologies across different physiological states in both humans and animals. Studies utilizing electroencephalography (EEG) or electrocorticography (ECoG) in humans demonstrate that RSNs derived from the power of multiple-site electrophysiological signals show similar spatial patterns to classic BOLD-derived RSNs such as the default-mode network (Hacker et al., 2017; Kucyi et al., 2018). In addition, simultaneous recordings of resting-state calcium and fMRI signals in awake rats show highly consistent spatial patterns between calcium- and BOLD-associated RSNs (Ma et al., 2022). Millisecond-timescale voltage-sensitive dye imaging reveals similar sensory-evoked and hemisphere-wide activity motifs represented in spontaneous activity in lightly anesthetized and awake adult mice (Mohajerani et al., 2013). All these data also well agree with the notion that the spatial patterns of RSNs are highly consistent with known functional systems and activation patterns observed in task-based studies (Biswal et al., 1995; Glasser et al., 2016; Hampson et al., 2002; Lowe et al., 1998; Smith et al., 2009), as well as patterns of structural networks (Andrews-Hanna et al., 2010; Greicius et al., 2009; Lowe et al., 2008). Taken together, previous studies and our data indicate consistent spatial patterns of RSNs can be derived from both electrophysiology and rsfMRI measurements, strongly suggesting that RSNs originate from neural activities.

Previous studies also demonstrate that RSNs are very likely constrained by axonal projections. Consistent sensory-evoked and hemisphere-wide activity motifs in mice revealed using voltage-sensitive dye imaging mentioned above are indeed defined by regional axonal projections (Mohajerani et al., 2013). Our previous work in awake rats also demonstrate spatial consistency between RSNs and anatomical connectivity patterns in thalamocortical networks (Liang et al., 2013). In the present study, we compared the RSNs (i.e. seedmaps) of M1 and ACC to their anatomical networks defined by the axonal projection patterns obtained from the database of Allen Brain Institute (Fig. S12, (Oh et al., 2014)). Our data show that RSNs revealed by either the BOLD or LFP signal well resemble the corresponding anatomical networks. Taken together, these data indicate that RSNs are most likely constrained by the backbone of structural connectivity. It needs to be pointed out that RSNs measured by functional connectivity can reflect both direct and indirect connectivity. Therefore, RSNs do not necessarily have identical spatial patterns as the corresponding anatomical networks (Honey et al., 2009).

In contrast to the seemingly strong LFP-BOLD relationship inferred from their high spatial correlations, we observed appreciably lower (but significant) temporal correlations between the two signals from the same recording sites (M1 and ACC). This result is corroborated by the finding that regressing out LFP power in any band has limited impact on RSN spatial patterns, indicating that the contribution of temporal variations of the LFP signal to RSN spatial patterns is minor. Despite the apparent discrepancy, these findings are in fact supported by several literature studies. In a well-controlled experiment, Winder et al. demonstrated weak but significant correlations between cerebral blood volume (CBV) changes measured by intrinsic optical imaging and spontaneous gamma-band LFP or multiunit activity in the barrel cortex in awake, head-fixed mice during rest (Winder et al., 2017). Importantly, they also observed persistent spontaneous fluctuations in CBV after blocking local neural spiking and glutamatergic input, as well as noradrenergic receptors, indicating that hemodynamic signal fluctuations may not necessarily reflect local ongoing electrophysiology activity (Winder et al., 2017). These data are in line with comparably low temporal correlations between gamma-band power and the rsfMRI signal in monkeys (Scholvinck et al., 2010; Shmuel and Leopold, 2008). Data published in our group also show similarly low temporal correlations between spontaneous calcium peaks, a measure of neural spiking activity, and the rsfMRI signal in awake rats (Ma et al., 2022). All these data collectively suggest that although the temporal correlation between electrophysiology and rsfMRI signals is significant, the effect size of this correlation might be small. Notably, our results are different from two earlier studies in isoflurane-anesthetized rats, which found that the LFP power in the primary somatosensory cortex was highly correlated with the BOLD fluctuation (Liu et al., 2011; Pan et al., 2011). This discrepancy might be attributed to the effect of brain-wide synchronization during burst suppression in deeply anesthetized states in those studies.

Why would electrophysiology and rsfMRI signals exhibit unmatched spatial and temporal correlations? Our model hypothesizes that the majority of the rsfMRI signal is driven by electrophysiology-invisible brain activities that are actively involved in NVC. Indeed, LFP is a composite signal that records various neural activities, such as synaptic potentials and voltage-gated membrane fluctuations, reflecting the input and local neural processing of a particular brain region. However, electrophysiology cannot measure activities from certain cell populations, while electrophysiology-invisible components can trigger vasoactive responses and significantly contribute to the rsfMRI signal. For instance, the electrical activity of nNOS neurons is not detectable by electrophysiology measurement because the nNOS neuron population is very small, but it strongly contributes to NVC. Chemogenetic or pharmacological stimulation of nNOS neurons causes vasodilation without detectable changes in LFP (Echagarruga et al., 2020). Furthermore, astrocytes, a type of glia cell, are known to coordinate communication between neurons and blood vessels and play a crucial role in NVC. Astrocytes contribute to the regulation of vessel tone by releasing signaling molecules such as ATP, arachidonic acid metabolites and nitric oxide (Ladecola and Nedergaard, 2007; Mulligan and MacVicar, 2004; Murphy et al., 1993). Additionally, astrocytes can alter the diameter of blood vessels by extending or retracting the endfeet wrapping around them (Mills et al., 2022; Niu et al., 2019). Optogenetically stimulating astrocytes in transgenic mice without affecting neurons led to a BOLD response, suggesting that astrocyte activity alone can cause changes in the BOLD signal, independent of neuronal activity (Takata et al., 2018). These studies collectively suggest some electrophysiology-invisible activities can significantly drive the rsfMRI signal. Exclusively identifying all electrophysiology-invisible sources that contribute to the rsfMRI signal is beyond the scope of this work. However, a key point is that as the resting state LFP and electrophysiology-invisible (and thus rsfMRI) signals reflect different components of brain activities, they can be minimally synchronized and display low temporal correlations. Another possible factor that can contribute to low BOLD-LFP temporal correlations is direct modulation of the vasculature from distant modulator nuclei (e.g. locus coerulues and/or basal forebrain). Some neuromodulators, such as norepinephrine (NE) (Bekar et al., 2012; Kim et al., 2016) and acetylcoline (Ach) (Sato and Sato, 1995), have vasoconstrictor/vasodilatory effects that are spatially targeted in distributed brain regions, and thus can modulate the brain-wide rsfMRI signal. However, these neural modulation effects may not be commensurately reflected from the electrophysiology signal, leading to low BOLD-LFP temporal correlations (see a schematic diagram in Fig. S13). On the other hand, as the signaling of LFP- and electrophysiology-invisible components in functional networks are constrained by the same anatomical connectivity structure (Liu et al., 2018a; Liu et al., 2018b), the LFP- and BOLD-derived RSN spatial patterns can be highly similar and thus have high spatial correlations.

Our model can potentially also explain high spatial and temporal correlations when brain activation is evoked by external stimulation (Winder et al., 2017). At the evoked state, both LFP (and spiking activity) and electrophysiology-invisible components are temporally modulated by the same external stimulation paradigm, leading to both high temporal and high spatial correlations between fMRI and electrophysiology signals.

## Summary

Our study revealed that BOLD-based RSNs can be reliably derived by the electrophysiology signal, but they might be dominantly contributed by the electrophysiology-invisible signal. This study provides a new interpretation of RSNs. However, this new concept of RSN signaling does not in any way argue against the importance of BOLD-based RSNs and the rsfMRI method. In fact, it makes fMRI even more important than previously thought because it might provide a new signal that traditional electrophysiology measures cannot provide.

## Methods and Materials

### Animals

All experiments in the present study were approved by and conducted in accordance with guidelines from the Pennsylvania State University Institutional Animal Care and Use Committee (IACUC). Adult male Long-Evans rats weighing 300–500g were obtained from Charles River Laboratory (Wilmington, MA). Animals were housed in Plexiglas cages with food and water given ad libitum. The ambient temperature was maintained at 22–24°C under a 12h light :12h dark cycle.

### Surgery

MR-compatible electrodes were implanted in animals with aseptic stereotaxic surgeries. The rat was briefly anesthetized with isoflurane before receiving intramuscular injections of ketamine (40 mg/kg) and xylazine (12 mg/kg). Baytril (2.5mg/kg) and long-acting buprenorphine (1.0mg/kg) were administered subcutaneously as antibiotics and analgesics, respectively. The animal was then endotracheal incubated and ventilated with oxygen using the PhysioSuite system (Kent Scientific Corporation). Body temperature was monitored and maintained at 37 °C with a warming pad placed underneath the animal (PhysioSuite, Kent Scientific Corporaition). Heart rate and SpO_2_ were continuously monitored using a pulse oximetry (MouseSTAT^®^ Jr, Kent Scientific Corporation) throughout the surgery. After performing craniotomies over the right ACC (coordinates: anterior/posterior +1.5, medial/lateral +0.5, dorsal/ventral −2.8) and the left M1 (coordinates: anterior/posterior +3.2, medial/lateral +3, dorsal/ventral −2.8), two MR-compatible electrodes (MRCM16LP, NeuroNexus Inc) were carefully implanted into the ACC and M1, respectively. The reference and grounding wires from each electrode were wired together and connected to one of the two silver wires placed in the cerebellum. At last, the skull was sealed with dental cement. After surgery, the animal was returned to the homecage and allowed to recover for at least one week before any experiment.

### Acclimation for awake imaging

Rats were restrained using a custom-designed restrainer during awake imaging sessions. To minimize stress and motion during the imaging process, animals underwent a 7-day acclimation procedure to the restrainer as well as the MRI environment and scanning noise. The duration of the acclimation procedure was gradually increased from 15 min on the first day to 60 min on days 4–7 days (i.e., 15 min on day 1, 30 min on day 2, 45 min on day 3, and 60 min on days 4–7). More details of the acclimation procedure can be found in previous publications from our laboratory (Liang et al., 2012) and other research groups (Bergmann et al., 2016; Chang et al., 2016).

### Simultaneous rsfMRI and electrophysiology recordings

All rsfMRI experiments were conducted on a 7T Bruker 70/30 BioSpec system running ParaVision 6.0.1(Bruker, Billerica, MA) using a homemade single loop surface coil at the *high field MRI facility* at the Pennsylvania State University. During each fMRI session, T2*-weighted rsfMRI images covering the entire brain were obtained using an echo planar imaging sequence with the following parameters: repetition time (TR) = 1 s; echo time (TE) = 15 ms; field of view = 3.2 × 3.2 cm^2^; matrix size = 64 × 64; slice number = 20; slice thickness = 1mm; volume number = 1200. Five to ten scans were repeated within each session. T2-weighted anatomical images were also acquired using a rapid acquisition with relaxation enhancement (RARE) sequence with the following parameters: TR = 3000 ms; TE= 40 ms; field of view = 3.2 × 3.2 cm^2^; matrix size = 256 × 256; slice number = 20; slice thickness = 1 mm; repetition number = 6.

Six rats with two electrodes implanted in the ACC and M1 were imaged in both awake and lightly sedated states in separate fMRI sessions. Two additional rats with an electrode only implanted in the ACC were imaged in the light sedation state. For both states, animals were restrained throughout the imaging session. In the light sedation state, the animal was sedated with the combination of low-dose dexmedetomidine (initial bolus of 0.05 mg/kg followed by a constant infusion at the rate of 0.1 mg·kg^−1^·h^−1^) and low-dose isoflurane (0.3%). Artificial tears were applied to protect the animal’s eyes from drying out. Body temperature was maintained at 37°C using warm air and was monitored using a rectal thermometer.

Before imaging, the implanted electrodes were connected to MR-compatible LP16CH headstages and a PZ5 neurodigitizer amplifier (Tucker Davis Technologies (TDT) Inc, Alachua, FL). Electrophysiology recording began 10 min before rsfMRI data acquisition and continued until the end of the imaging session using a TDT recording system and an RZ2 BioAmp Processor (TDT Inc, Alachua, FL). The raw, unfiltered electrophysiology signal was sampled at 24414 Hz and stored using the TDT Synapse software on a WS8 workstation.

### rsfMRI and electrophysiology data preprocessing

All data preprocessing and analysis were performed using MATLAB (Mathworks, Natick, MA). First, the movement of each rsfMRI volume was estimated using the framewise displacement (FD). For the awake imaging data, volumes with FD > 0.1 mm and their adjacent preceding and following volumes were removed. If > 25% of volumes in a scan were scrubbed, the entire scan was excluded from further analysis. For rsfMRI data collected in the lightly sedated state, scans with any volume that had FD > 0.1 mm were removed from further analysis. Subsequently, data were preprocessed using the following steps: co-registration to a defined atlas, motion correction (SPM12), spatial smoothing using a Gaussian kernel (FWHM = 0.75 mm), voxelwise nuisance regression with the regressors of motion parameters as well as signals from the white matter and ventricles, and the global brain signal, and, lastly, bandpass filtering (0.01–0.1 Hz).

Raw electrophysiology data were preprocessed to remove the MR interference using a template regression method as previously described (Tu and Zhang, 2022). Briefly, the raw electrophysiology signal for each scan was first aligned to the corresponding rsfMRI scan, and segmented for each imaging slice based on the starting time of the scan. Next, an MRI interference template for each rsfMRI slice acquisition was obtained by averaging the electrophysiology data across all slices from all rsfMRI volumes. The template was further aligned to the electrophysiology data for each slice acquisition using cross correlation and was then linearly regressed out from the raw electrophysiology data. In addition, a series of notch filters for harmonics of the power supply (60 Hz and multiples of 60 Hz) and slice acquisition (20 Hz and multiples of 20 Hz) were applied to further denoise the data.

### Data analysis

The LFP power was obtained by bandpass filtering preprocessed electrophysiology data in the frequency range of 0.1 – 300 Hz. Based on the conventional LFP band definition (delta: 1–4 Hz, theta: 4–7 Hz, alpha: 7–13 Hz, beta: 13–30 Hz, gamma: 40–100 Hz) (Lu et al., 2016; Zhang et al., 2020), the LFP band power was computed using the MATLAB function *spectrogram* with a window size of 1 s and a step size of 0.1 s. To investigate the relationship between the LFP and rsfMRI signals, the time course of band-specific LFP power was convolved with a hemodynamic response function (HRF, p = [4 4 1 1 6 0 32] for function *spm_hrf)* to generate the corresponding LFP-predicted BOLD signal. The HRF used was specific to rodents with a shorter onset time and time-to-peak as a faster HRF was reported in rats relative to humans (Tong et al., 2019). The temporal relationship between the LFP and fMRI signal was quantified using the Pearson correlation between the HRF-convolved LFP band power and the regionally averaged rsfMRI time course from voxels surrounding the implanted electrode for each site. To examine the potential impact of HRF used, we calculated the BOLD-gamma power correlation using different HRFs with various response delays, ranging from 2 sec to 8 sec with the increment of 0.25 sec, as well as different undershoot delays ranging from 2 sec to 12 sec with the increment of 0.5 sec.

The LFP-derived spatial correlation maps for the M1 and ACC were respectively generated by computing voxel-wise Pearson correlations between each HRF-convolved LFP band power and brain-wide rsfMRI signals. The seedmaps for the M1 and ACC were respectively obtained by calculating the voxel-wise Pearson correlations between the regionally averaged rsfMRI time course for each seed and the rsfMRI signals of all brain voxels.

To determine the contribution of LFP power to BOLD-based RSFC, we removed the LFP powers from voxel-wise fMRI signals and then recalculated RSNs. Given that the gamma signal might be the most related to the rsfMRI signal, the time course of gamma-band power (convolved with HRF) was linearly regressed out from rsfMRI signals of all brain voxels. To examine the potential contributions of other LFP bands, all five LFP band powers (each convolved with HRF) were “softly” removed from voxel-wise rsfMRI signals, meaning only the unique components in five bands were regressed out but the shared components were maintained. Specific details of soft regression can be found in (Griffanti et al., 2014). This method can avoid ‘over regression’ when multiple regressors are involved particularly when regressors are correlated between themselves. Lastly, we removed rsfMRI volumes corresponding to peaks in the M1/ACC gamma power. The seedmaps of M1 and ACC were recalculated and compared before and after removing the LFP signal.

### Simulation

We simulated two fixed signals with the true Pearson correlation of 0.95. The first signal was generated using MATLAB function *rand* with 10000 data points. The second signal with a defined correlation with the first signal (i.e. 0.95) was obtained based on the equation below:

$$B=A*Corr+\sqrt{1-{Corr}^{2}}*N(0,1)$$

in which A represents the first signal, Corr is the desired Pearson correlation coefficient, and N(0,1) represents random values with the mean equal to 0 and standard deviation equal to 1. For each signal, random noise was added to achieve a contrast-to-noise ratio (CNR) ranging from 0.1 to 5 with the step size of 0.1. CNR was quantified by the standard deviation of the signal over the standard deviation of the noise. This process was repeated 159 times (equal to the # of scans in the present study). The noise-added signals were resampled to either 1200 or 6157 data points, which corresponded to the total number of time points used to calculate temporal correlations and total number of brain voxels used to calculate spatial correlations, respectively, in our study. Pearson correlations between the resampled signals either based on the averaged signals from all 159 trials or on individual trials were calculated.
